# Intestinal Obstruction Removed by Position, Distensile Enema and Gas

**Published:** 1875-08

**Authors:** A. B. Copeland

**Affiliations:** Valley Plains, Ga.


### Intestinal Obstruction Removed by Position, Distensile Enema and Gas.

By A. B. COPELAND, M.D., Valley Plains, Ga.

In company with Dr. T. S. Mitchell, I visited the following case, which we found to present a facial expression of great constitutional disturbance. There was present the usual symptoms of intestinal obstruction, including stercoraceous vomiting. The patient had taken cathartic medicine by wholesale. We at once placed him in an inclined position at an angle of about 45°, the head downwards. Several times we plied the distensile enema, employing each time two gallons of warm water, and forcing the retention of it for some minutes at each time by compressing a napkin upon the anus. The efforts failing to relieve, we next threw in one gallon of the water, which was retained, and in half an hour a second gallon was thrown in and forcibly retained by compression for one hour longer, the patient being kept in the same inclined position. In response to his urgent complaints he was now allowed to get up to stool, but passed nothing save the water injected.

The thought now occurred to us that if we could renew the same distension from below, and secure at the same time a like distension of the intestinal tube from above the obstruction, that we would likely succeed in its removal. Accordingly two drachms of the bicarbonate of soda, in solution, was administered by the mouth, and soon followed by a solution of one drachm of tartaric acid. Now immediately inverting the patient again, as before, we quickly threw into the bowels two gallons of the warm water. Vomiting now came on, the carbonic acid gas bursting from the mouth, and being placed upon the stool a large fecal evacuation occurred with relief to all the symptoms. To satisfy myself of the full permeability of the canal, I returned the following day, and, injecting a half pint of castor oil into the rectum, followed it with large injection of warm water until vomiting occurred. The patient distinctly tasted the castor oil in the vomited fluid, and the oil globules were abundantly floating upon the ejected liquid.

